# Azoxystrobin-Induced Physiological and Biochemical Alterations in *Apis mellifera* Workers of Different Ages

**DOI:** 10.3390/insects16050449

**Published:** 2025-04-24

**Authors:** Xinle Duan, Wenlong Tong, Bingfang Tao, Huanjing Yao, Manqiong Xiong, Huiping Liu, Shaokang Huang, Jianghong Li

**Affiliations:** 1 College of Bee Science and Biomedicine, Fujian Agriculture and Forestry University, Fuzhou 350002, China; wenlongtong@sina.cn (W.T.); bingfangtaofafu@outlook.com (B.T.); yhjonline@outlook.com (H.Y.); mqxiong.dingle@outlook.com (M.X.); skhuang@fafu.edu.cn (S.H.); leejh@fafu.edu.cn (J.L.); 2 State Key Laboratory of Ecological Pest Control for Fujian and Taiwan Crops, Fujian Agriculture and Forestry University, Fuzhou 350002, China; liuhuipingfafu@outlook.com; 3 Fujian Honeybee Biology Observation Station, Ministry of Agriculture and Rural Affairs, Fuzhou 350002, China; 4 College of Plant Protection, Fujian Agriculture and Forestry University, Fuzhou 350002, China

**Keywords:** *Apis mellifera*, azoxystrobin, survival, enzyme activity, gene expression

## Abstract

Fungal diseases of agricultural crops cause severe economic losses to the growers. For the control of these diseases, azoxystrobin is one of the recommended fungicides. This fungicide is systemic in action and is expected to reach the floral part of the treated crop and its residue in the pollen and nectar, the natural food sources of honey bees, which could be collected and fed on by honey bees, thus affecting their health. The purpose of this study was to determine the physiological and chemical changes caused by this fungicide in honey bee workers (*Apis mellifera* L). Workers of this honey bee at 1, 8, and 21 days old were treated with 125, 167, and 250 mg/L concentrations of azoxystrobin for seven days; their survival rates, activities of carboxylesterase (CarE), glutathione S-transferases (GSTs), cytochrome P450 enzyme (CYP450), catalase (CAT), and superoxide dismutase (SOD) enzymes, and the expression levels of immune (*Aba*, *Api*, *Def1*, and *Hym*) and nutrition genes (*Ilp1*, *Ilp2*, and *Vg*) were detected. Our findings revealed that azoxystrobin affected the survival of workers, particularly 1- and 21-day-old workers, who responded to azoxystrobin stress with increased activities of detoxification and protective enzymes, which might have physiological costs. Additionally, azoxystrobin affected the expression of immune and nutrition genes, with a decreased expression trend in 21-day-old workers compared to the 1- and 8-day-old workers, leading to reduced resistance to external stressors and increased mortality rates. These findings provide important insights into the adverse effects of azoxystrobin on workers of different ages and emphasize the potential risks of this chemical to colony stability and individual health. This study recommends an urgent ban on such a harmful fungicide being used for fungi control in agriculture, especially during plant flowering.

## Simple Summary

This study investigates the effect of azoxystrobin on the survival and physiological functions of workers of different ages (1-, 8-, and 21-day-old). Honey bees are vital pollinators, and their declining populations pose an ecological and economic threat. While azoxystrobin is widely used in agriculture, its effects on non-target organisms such as bees are not fully understood. To fill this knowledge gap, the effects of azoxystrobin on workers’ survival rates, detoxification and protective enzyme activities, and the expression of immune and nutrition-related genes were determined. The results indicated that azoxystrobin exposure significantly reduced survival rates, particularly in younger (1-day-old) and older (21-day-old) workers, and altered enzyme activities and gene expression levels. These findings highlight the age-dependent sensitivity of workers to the fungicide, which is critical for developing strategies for fungicide usage to protect honey bee populations and ensure sustainable agricultural practices. By optimizing fungicide use and taking into account the physiological state of workers, adverse effects on these important pollinators can be mitigated and ecological balance maintained.

## 1. Introduction

Plant diseases not only cause a reduction in crop yields but also, to a certain extent, threaten the safety of the products for human consumption [1,2]. Fungicides provide hard-to-replace benefits in plant disease control to ensure the security of the global food harvest and production [3,4,5]. The toxicity of fungicides is evaluated during their development and registration, and evaluation results show that most fungicides have low oral toxicity or contact toxicity for bees [6]. However, low toxicity does not necessarily mean safe or nontoxic, and many fungicides are not friendly to pollinator bees [4,7,8,9]. For example, systemic fungicides are absorbed by plants and provide systemic protection, which can lead to residues in their pollen and nectar and foragers being directly or indirectly exposed to fungicides [10,11]. These contaminated foods are collected by foragers and consumed by other bees in the colony, affecting individual and colony health [12,13,14]. With this in mind, the present study was conducted on the ecotoxicity of fungicides on the physiological and biochemical response of honey bees.

Honey bees (*Apis mellifera*), as keystone pollinators, are vital to plant reproduction, agricultural productivity, and biodiversity [15,16]. They contribute substantially to the pollination of global flowering plants and agricultural production [17,18,19,20]. However, their populations have declined sharply worldwide, notably in China, the USA, Brazil, and Argentina, threatening ecosystems and entomophilous plant survival, with climate change, intensive agriculture, parasites, and pathogens exacerbating colony health issues [17,21,22,23,24,25]. Among these stressors, pesticide exposure is a critical driver of pollinator decline, disrupting worker development, physiology, and behavior [12,13,26]. Boscalid (0.5 ng/μL) reduced the survival and reproduction of immature queens [27]. Tebuconazole (34.625 mg a.i/L) altered the activities of carboxylesterase (CarE), cytochrome P450 enzyme (CYP450), and the gene expressions of *apidaecin*, *CYP4G11*, and *CYP6AS14* in 3-day-old workers [28]. Pyraclostrobin (250 mg/L) induced oxidative damage in workers by disturbing the activities of glutathione S-transferases (GSTs), superoxide dismutase (SOD), and catalase (CAT) [16], and damaged fat body and pericardial cells [29]. Pristine^®^ (23 ppm) impairs post-ingestive glucose signals and learning performance [30,31]. Chlorothalonil (10 μg/L) and difenoconazole (0.27 mg/L) disrupt the structure and function of the gut microbial community and detoxification/immunity pathways [32,33].

Azoxystrobin, a prominent strobilurin-class fungicide, operates by inhibiting mitochondrial respiration in fungi, thereby disrupting their energy synthesis [2]. According to an EFSA report, the commercial formulation of azoxystrobin (250 g/L) is recommended for disease control in cereals and Brassica vegetables by spraying after being diluted 1000–4000 fold [34]. Due to its broad-spectrum activity and efficiency, azoxystrobin has been widely used to control fungal diseases in rice, wheat, rape, and other nectar plants [35,36]. However, some studies have indicated the potential adverse effects of azoxystrobin on non-target organisms [37,38]. It could cause nuclear abnormalities and DNA damage in *Pethia conchonius*, and has a dose- and time-dependent relationship with exposure concentrations (0.025, 0.0514, and 0.103 mg/L) [37], also inducing reactive oxygen species (ROS) and suppressing the immune ability of *Eisenia fetida* with exposure doses (2 and 2.5 μg/cm^2^) [38]. Also, this systemic fungicide is absorbed by plants and translocated to their pollen and nectar. Honey bees may encounter azoxystrobin directly during spraying or indirectly via contaminated pollen and nectar, potentially leading to colony exposure [23,34,39]. While classified as a low bio-concentration potential and moderate-risk substance to bees [34], azoxystrobin (250 µg/µL) could lead to the higher mortality of bumble bees [40] and cytoplasm vacuolization, cell fragmentation of the midgut [41], and transcriptional alterations of oxidative phosphorylation and metabolism [42] in honey bees.

Honey bees are eusocial insects characterized by a pronounced division of labor, with workers of different ages performing specialized tasks as super-organisms [43]. For instance, nurse bees (approximately eight days old) utilize the pollen to produce royal jelly, whereas foragers (approximately 21 days old) primarily collect pollen and nectar [43,44]. These roles are crucial for the health of the colony, and if any of the specialized tasks were threatened by external stresses, such as environmental changes, pathogens, parasites, and pesticides, the entire colony would also be affected [16].

The age-related division of labor in workers is intrinsically linked to the dynamic shifts in their physiological and biochemical profiles [45], which may determine their sensitivity to fungicides. Building on these findings, we hypothesize that azoxystrobin could also disrupt the normal physiological and biochemical metabolisms of workers of different ages, potentially increasing their sensitivity to azoxystrobin. To test this hypothesis, an experimental framework was designed to with the following aims: (i) to investigate the age-specific survival rates of workers (1-, 8-, and 21-day-old) exposed to field-relevant concentrations of azoxystrobin; (ii) to assess oxidative stress and detoxification responses by measuring the activities of catalase (CAT), superoxide dismutase (SOD), carboxylesterase (CarE), glutathione S-transferases (GSTs) and cytochrome P450 enzyme (CYP450) enzymes across age groups and treatments; (iii) to evaluate molecular mechanisms underlying sensitivity differences by analyzing expression levels of immunity genes, *abaecin* (*Aba*), *apidaecin* (*Api*), *defensin1* (*Def1*), and *hymenoptaecin* (*Hym*), and nutrient genes, *insulin-like peptide* 1 (*Ilp1*) and 2 (*Ilp2*), and *vitellogenin* (*Vg*), in azoxystrobin-exposed workers.

## 2. Materials and Methods

### 2.1. Honey Bee (A. mellifera) Rearing

The methods of rearing and collecting workers of the same age referred to our previous study [16]. To keep the sensitivity of the honey bees to pesticides, standard beekeeping practices and strict precautions were taken at least one month before the experiments to ensure that the workers were not contaminated with other chemicals or pesticides [9]. Briefly, frames with capped broods were randomly selected from honey bee colonies and then moved into an artificial climate incubator (34 ± 5 °C, darkness, and 75% RH) for eclosion. When workers began to emerge, the newly emerged bees (0–24 h old, considered as 1 day old) were collected, their front thoracic dorsal plates were marked in red, and they were then reintroduced into the colonies until their use for the exposure experiments. The workers with markers were collected on the 7th day and were considered to be nurse bees (8 days old), and the workers collected on the 19th day were considered forager bees (20 days old). These workers of different ages were reared on 50% sucrose solution (*w*/*v*) and fresh pollen for the subsequent azoxystrobin exposure.

### 2.2. Fungicide

Considering realistic sales and usage, 250 g/L azoxystrobin SC was purchased from Syngenta Crop Protection (Nantong) Co., Ltd. (Nantong, China), and employed to assess its ecotoxicological effects on workers of different ages. The commercial formulation of azoxystrobin was diluted with a sucrose solution (50%, *w*/*v*) to obtain different solutions of azoxystrobin with concentrations of 125 mg/L, 167 mg/L, and 250 mg/L according to the dilutions recommended in the instruction manual, and then these solutions were stored at −4 °C and used up within 7 days.

### 2.3. Azoxystrobin Treatment

The 1-, 8-, and 21-day-old workers were collected from the colonies and then quickly transferred to an incubator. All of them were starved for 6 h before exposure to azoxystrobin. Workers of the same age were assigned to four groups: control (CK), 125, 167, and 250 mg/L azoxystrobin treatment groups. Each group consisted of 5 replicates of 25 workers, for a total of 125 workers per group. The workers in the azoxystrobin treatment groups were fed daily with prepared syrup containing azoxystrobin, while those in the CK received only syrup without azoxystrobin. These workers were checked daily, and we removed the dead individuals and recorded the number of deaths for the survival statistics. During the azoxystrobin exposure treatment, all workers had access to food ad libitum, and the fresh syrup containing azoxystrobin was changed daily. The oral exposure duration for all the workers was set at 7 days, meaning that 1-day-old workers would become 8 days old. The live workers were randomly collected from each group after exposure for the determination of enzyme activity and gene expression.

### 2.4. Biochemical Tests on Enzyme Activity

Five honey bees were randomly selected from each treatment for enzyme extracts. Their whole bodies were mixed with a specific buffer at 4 °C and then homogenized using a high-speed TissueLyser (Scientz Biotechnology Co., Ltd., Ningbo, China) at 60 Hz for 1 min. The initial enzyme extracts were centrifuged (4 °C, 10 min, 12,000× *g*) and the supernatant was retained on ice for activity determinations. The respective commercial assay kits were used for CAT, SOD, CarE, and GSTs activity detection (Comin Biotechnology Co., Ltd., Hangzhou, China). CYP450 activity was evaluated with an ELISA kit from J&L Biological Technology Co., Ltd. (Shanghai, China). These experiments were strictly conducted according to the kit instructions and the assays were carried out in triplicate to ensure the reliability of the results. The calculation method of enzyme activity refers to the recommended method in the kit instructions.

### 2.5. Gene Expression Tests for Immunity and Nutrition Metabolism

The TRIzol Reagent (Invitrogen, Waltham, MA, USA) was used to extract the total RNA of the adult workers. Following gel electrophoresis and absorbance determination, the RNA samples underwent cDNA synthesis using the PrimeScript™ RT reagent kit (TaKaRa, San Jose, CA, USA), following its instructions, and then the cDNA samples were stored at −20 °C for further detection. *β-actin* served as the internal control gene, and detailed primer information for the *β-actin*, nutrient, and immune genes is provided in Table S1. The quantitative RT-PCR was performed according to standard protocols and cycling conditions; the qPCR system (10 µL) included ROX Reference Dye II (50×), 0.2 μL; TB Green Premix Ex Taq II (2×), 5 μL; cDNA, 1 μL; each forward and reverse primer, 0.4 μL; and ddH_2_O, 3 μL. The thermal cycling conditions were 95 °C for 30 s, 40 cycles of denaturation at 95 °C for 5 s, 60 °C for 30 s, and a final melt curve step was run from 60 to 95 °C for 10 s at 1 °C increments to check for non-specific amplification. At least three technical and biological triplicates were performed for the detection of gene expression. The mRNA expression levels of the target genes were quantified by the 2^−ΔΔCt^ method [46].

### 2.6. Statistical Analysis

GraphPad Prism 10 (GraphPad Software, Boston, MA, USA) was used for statistical analysis and figure drawing. The Log-rank (Mantel–Cox) test for the Kaplan–Meier method was used to determine the significance of the differences in survival rate, and one-way ANOVA with Tukey’s multiple comparison tests was also used for statistically significant differences in enzyme activity and gene expression. The significance level for the data was set at *p* < 0.05.

## 3. Results

### 3.1. Azoxystrobin Decreased Survival Rate of Workers

**Figure 1:**
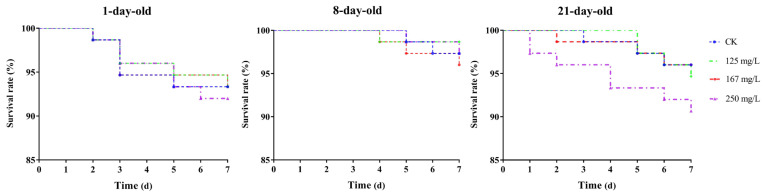
Effect of azoxystrobin on survival rates of worker bees of different ages.

The survival rate of the workers of different ages (1-, 8-, and 21-day-old) in all the azoxystrobin treatment groups decreased continuously with exposure time (Figure 1). Compared to 8-day-old workers, the 1- and 21-day-old workers had a poor tolerance to azoxystrobin, and the survival rates of them in each treatment group were lower than that of the 8-day-old workers. However, no significant differences in the survival rate of the workers (1-, 8-, and 21-day-old) were found between different the concentration treatment groups and the CK (Log-rank (Mantel–Cox) test, for 1-day-old workers, *x^2^* = 0.1471, *df* = 3, *p* = 0.9856; for 8-day-old workers, *x^2^* = 1.018, *df* = 3, *p* = 0.7970; for 21-day-old workers, *x^2^* = 2.783, *df* = 3, *p* = 0.4262).

### 3.2. Azoxystrobin Interfered with Detoxification Enzymes Activities of Workers

**Figure 2:**
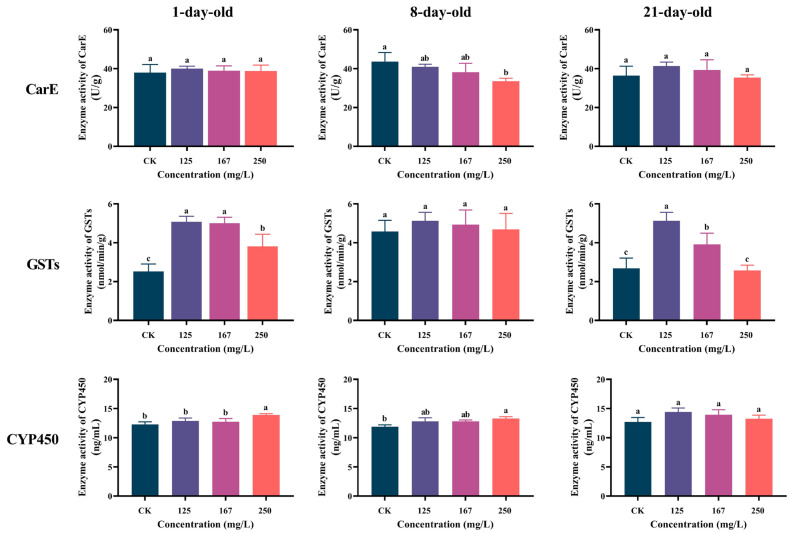
Effect of azoxystrobin on activities of detoxification enzymes (CarE, GSTs and CYP450) in worker bees of different ages. Data on concentration in figure expressed as mean ± SE (standard error). Different letters mean significant differences among different exposure treatments (Tukey’s multiple comparison tests, *p* < 0.05).

After being exposed to azoxystrobin, the CarE activity of the 1-day-old workers was elevated compared to the CK, although the difference was not statistically significant (one-way ANOVA, F(3,12) = 0.3404, *p* = 0.7966; Figure 2). However, the CarE activity of the 8-day-old workers was decreased in the treatment groups compared to that of the control, particularly in those treated with 250 mg/L azoxystrobin (F(3,9) = 5.393, *p* = 0.0178). In the 21-day-old workers, CarE activity exhibited low-concentration induction (125 and 167 mg/L) and high-concentration inhibition (250 mg/L) in response to azoxystrobin exposure (F(3,11) = 1.682, *p* = 0.2281). GST activity was increased in both the 1- and 8-day-old workers with azoxystrobin stress, especially in the 1-day-old workers (F(3,9) = 27.82, *p* < 0.0001), while the GST activity of the 21-day-old workers was significantly increased in the 125 and 167 mg/L groups (F(3,12) = 26.49, *p* < 0.0001). In these workers of different ages, their CYP450 activities were increased; especially, those of the 1- (F(3,13) = 7.745, *p* = 0.0032) and 8-day-old workers (F(3,12)= 6.073, *p* < 0.0001) were significantly increased in the 250 mg/L group.

### 3.3. Azoxystrobin Altered Protective Enzymes Activities of Workers

**Figure 3:**
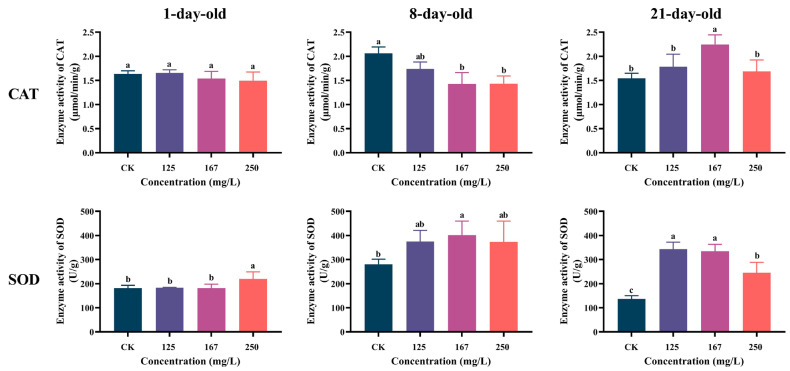
Effect of azoxystrobin on activities of protective enzymes (CAT and SOD) in worker bees of different ages. Data on concentration in figure expressed as mean ± SE (standard error). Different letters mean significant differences among different exposure treatments (Tukey’s multiple comparison tests, *p* < 0.05).

Azoxystrobin inhibited CAT activity in both 1- and 8-day-old workers, but no significant difference between the CK and treatment groups for the 1-day-old workers was found (one-way ANOVA, F(3,12) = 1.487, *p* = 0.2677; Figure 3), while the CAT activity of the 8-day-old workers was obviously decreased in the 167 and 250 mg/L groups (F(3,12) = 12.14, *p* = 0.0006). In the 21-day-old workers, the CAT activity increased in the azoxystrobin treatment groups, with a significant rise in the 167 mg/L group (F(3,12) = 6.663, *p* = 0.0025). The SOD activity in these workers increased with azoxystrobin exposure, but the effect varied with concentration and age. Among the groups, the SOD activity of the 21-day-old workers was significantly increased with azoxystrobin stress (F(3,11) = 43.40, *p* < 0.0001). This was not the case for the 1- and 8-day-old workers; the significant effect on 1-day-old workers was only in the 250 mg/L group (F(3,11) = 4.343, *p* = 0.0452), while for 8-day-old workers it was only in the 167 mg/L group (F(3,10) = 4.3470, *p* = 0.0489).

### 3.4. Azoxystrobin Disrupted Immune Response of Workers

**Figure 4:**
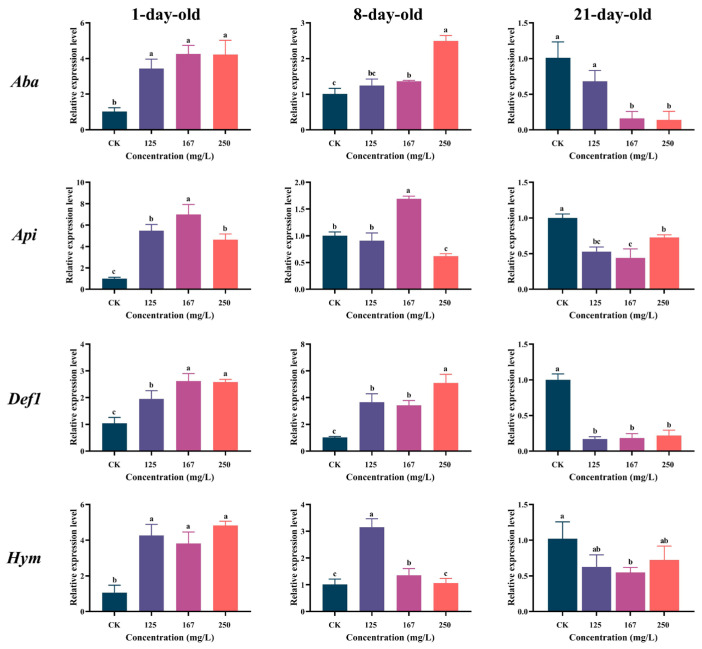
Effect of azoxystrobin on expression of immune genes (*Aba*, *Api*, *Def1* and *Hym*) in worker bees of different ages. Data on expression level in figure expressed as mean ± SE (standard error) and different letters mean significant differences among different exposure treatments (Tukey’s multiple comparison tests, *p* < 0.05).

The expression levels of four immune genes (*Aba*, *Api*, *Def1* and *Hym*) in the 1-day-old workers exhibited a significant upregulation trend in response to azoxystrobin pressure (one-way ANOVA: for *Aba*, F(3,8) = 23.36, *p* = 0.0003; for *Api*, F(3,9) = 50.50, *p* < 0.0001; for *Def1*, F(3,9) = 33.91, *p* < 0.0001; for *Hym*, F(3,8) =32.26, *p* < 0.0001; Figure 4). Among them, the expression levels of *Aba*, *Api*, and *Def1* were highest in the 167 mg/L group, and the highest expression level of *Hym* was in the 250 mg/L group. Conversely, the expression levels of these immune genes in the 21-day-old workers were downregulated with azoxystrobin exposure, especially those of *Api* and *Def1* (for *Api*, F(3,8) = 35.15, *p* < 0.0001; for *Def1*, F(3,13) = 134.2, *p* < 0.0001). Azoxystrobin exposure led to an upregulated expression of *Aba*, *Def1*, and *Hym* with a different expression pattern in the 8-day-old workers, with the expression levels of *Aba* (F(3,11) = 81.49, *p* < 0.0001) and *Def1* (F(3,8) = 36.76, *p* < 0.0001) being the most highly expressed in the 250 mg/L group, while *Hym* was most highly expressed in the 125 mg/L group (F(3,10) = 56.70, *p* < 0.0001). Meanwhile, the expression level of *Api* was significantly upregulated in the 167 mg/L group but significantly downregulated in the 250 mg/L group (F(3,9) = 105.9, *p* < 0.0001).

### 3.5. Azoxystrobin Perturbed Nutrition Metabolism of Workers

**Figure 5:**
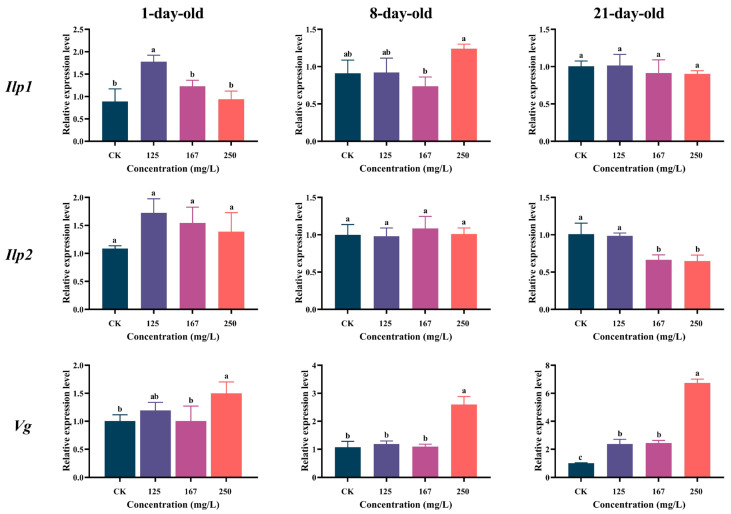
Effect of azoxystrobin on expression of nutrition genes (*Ilp1*, *Ilp2* and *Vg*) in worker bees of different ages. Data on expression level in figure expressed as mean ± SE (standard error) and different letters mean significant differences among different exposure treatments (Tukey’s multiple comparison tests, *p* < 0.05).

The expression levels of three nutrition genes (*Ilp1*, *Ilp2*, and *Vg*) in the 1-day-old workers were increased by azoxystrobin stress. The expression of *Ilp1* was significantly increased only in the 125 mg/L group (one-way ANOVA, F(3,9) = 13.76, *p* = 0.0010; Figure 5) and that of *Vg* was only increased in the 250 mg/L group (F(3,8) = 5.025, *p* = 0.0302), while the expression of *Ilp2* was upregulated, but no statistically significant difference was observed (F(3,8) = 3.362, *p* = 0.0756). Both *Ilp1* and *Ilp2* were downregulated in the 21-day-old workers, and their levels gradually decreased with the increasing concentrations of azoxystrobin treatment (for *Ilp1*, F(3,8) = 0.6714, *p* = 0.5932; for *Ilp2*, F(3,8) = 15.12, *p* = 0.0007). Azoxystrobin could induce the expression of *Vg* with a significant concentration effect (F(3,10) = 314.6, *p* < 0.0001). In the 8-day-old workers, the three nutrition genes were upregulated in the 250 mg/L group, but only *Vg* showed a significant difference compared to the CK (for *Ilp1*, F(3,10)= 6.593, *p* = 0.0098; for *Ilp2*, F(3,9) = 0.3983, *p* = 0.7576; for *Vg*, F(3,8) = 44.41, *p* < 0.0001).

## 4. Discussion

The population size and health status of workers are pivotal to colony sustainability. Adverse impacts on these critical factors may precipitate colony disorganization or even collapse, thereby threatening ecological stability and pollination services [16,47]. Our results demonstrate that azoxystrobin exposure impacts the survival rates, enzyme activities, and gene expression profiles of workers. These findings confirm the adverse effects of azoxystrobin on the survival and physiological responses of workers of different ages, highlighting the potential risks this chemical poses to colony stability and individual health.

Following the acute oral and contact toxicity testing methods recommended by the Organization for Economic Cooperation and Development (OECD), workers of *A. mellifera* were randomly divided into different groups and exposed to a single dose of azoxystrobin by feeding them a contaminated sucrose solution, or by topical application, and the LD_50_ values were calculated based on mortality in each treatment group. Both the acute oral and contact toxicity LD_50_ values of azoxystrobin to the workers were >200 μg/bee, indicating its low oral and contact toxicity to workers [34]. However, azoxystrobin has a negative impact on workers’ survival, and the progressive decline in survival rates with prolonged exposure time is particularly evident in the high-concentration treatment (250 mg/L, Figure 1), which is consistent with the age-dependent differences in the sensitivity of bees to strobilurin fungicide types, such as pyraclostrobin, in other studies [16]. This phenomenon may be related to changes in the midgut physiology induced by azoxystrobin, evidenced by histopathology and cytotoxicity [41], as well as the disruption of the intestinal microbial community [4].

Meanwhile, the lack of significant differences in survival rates between azoxystrobin-exposed groups and the CK group (*p* > 0.05) may be attributed to the relatively short exposure duration or the specific concentrations used [9,48]. Notably, the poor tolerance of the 1- and 21-day-old workers to azoxystrobin highlights the age-dependent variations in sensitivity, which are closely associated with the division of labor and physiological states of the workers [16,49]. In comparison to the 1- and 8-day-old workers, the 21-day-old workers responsible for the collection of food resources exhibit a markedly elevated likelihood and magnitude of exposure to pesticides within their environmental habitats, and azoxystrobin can disrupt their intestinal cells and microbiota, leading to a decrease in survival rate and exhibiting high sensitivity [41,42]. Moreover, the age-dependent differences in sensitivity underscore the importance of considering the developmental stage of workers when assessing the impacts of agricultural chemicals on bee colonies.

During the long-term process of evolution with the environment, insects are equipped with a sophisticated array of enzyme systems, including protective enzymes and detoxification enzymes, that work in tandem to mitigate the lethal effects of agricultural chemicals [50]. The protective enzymes play a crucial role in eliminating the toxic effects of intracellular free radical accumulation [51]. In most organisms, SOD and CAT form the first line of defense against ROS attack by acting directly or indirectly on ROS molecules, where SOD quickly transforms excess superoxide radicals (O_2_^−^) into hydrogen peroxide (H_2_O_2_) and then CAT breaks this down into water (H_2_O) and oxygen (O_2_) [51,52]. This would maintain free radicals at a low level of dynamic equilibrium, thereby preventing damage to the organism, and they are commonly used as indicators of insect physiological status [52]. Azoxystrobin, an exogenous toxic substance with cyclic chemical compounds, has been shown to induce severe oxidative damage in earthworms [53], zebrafish [54], and other organisms, even leading them to death [37]. The increased SOD activities in workers of different ages indicates that azoxystrobin induces oxidative stress in the workers (Figure 3). Simultaneously, the elevated SOD activity catalyzes the conversion of superoxide anion radicals, thereby providing protective effects against oxidative damage in workers [55]. CAT activity in the 8-day-old workers was obviously reduced, which may be due to the production of a large amount of ROS under the azoxystrobin stress affecting its activity [56]. This in turn leads to the accumulation of H_2_O_2_ and causes oxidative stress [23]. Meanwhile, the lower mortality rate of the 8-day-old workers suggests that oxidative stress would trigger other defense pathways in workers to neutralize ROS [57].

As crucial detoxification enzymes in insects, CarE, GSTs, and CYP450 can metabolize exogenous toxic substances to water-soluble, excretable metabolites, and insects adjust their activity to maintain normal physiological metabolisms and enhance stress resistance and drug tolerance [50,51,58,59,60]. In detoxification reactions, CarE and CYP450 play an important role in the primary metabolism, in that they modify toxic compounds or structures by oxidation or hydrolysis reactions, rendering them unable to interact with lipophilic target sites and transforming them into water-soluble, excretable forms [61,62]. GSTs is a multifunctional cytosolic enzyme, and these GSH-dependent enzymes could catalyze the conjugation of electrophilic toxic compounds with glutathione (GSH), increasing their water solubility, promoting their excretion, and also directly participating in detoxification by binding and sequestering toxins (such as pyrethroids and ROS) [63]. Here, the varying activities of CarE, GSTs, and CYP450 in workers could mitigate the harmful influence of azoxystrobin, and these variations exhibit distinct patterns among workers (Figure 2). CarE has been shown to degrade strobilurin fungicides effectively [64]. In our study, there was no significant change in CarE activity in the 1- and 21-day-old workers within the azoxystrobin-exposed groups. However, CarE activity in the 8-day-old workers decreased significantly with azoxystrobin concentration. This suggests that workers encounter problems in the primary metabolism of azoxystrobin, and this may relate to the over-accumulation of ROS induced by azoxystrobin, which activates a variety of intracellular signaling pathways, leading to changes in the intracellular metabolic environment, thereby affecting the activity of CarE [16,64]. The CYP450 activity for all the workers was increased in all azoxystrobin treatment groups, indicating that workers can effectively carry out the primary metabolism of toxic substances by increasing the activity of CYP450, even though the activity of CarE was inhibited, and this demonstrates that CYP450 is crucial for the primary metabolism of azoxystrobin by workers [65]. Meanwhile, an increase in GST activity can help workers effectively execute the secondary metabolism of toxic substances, which enhances the water solubility of the primary metabolites produced by CarE and CYP450, thereby reducing the negative impact of toxic substances on organisms [66]. Additionally, as an antioxidant substance, GSTs can solve the thorny problem of ROS accumulation in workers which is induced by azoxystrobin [49], thereby compensating for the deficiency of decreased protective enzyme activity [16]. Enzyme activity assays show that workers coordinate protective and detoxification enzyme activities to handle azoxystrobin stress, suggesting an adaptive response to oxidative stress and maintaining normal physiological functions in their bodies as much as possible, but this causes certain physiological costs for the workers [16,23,67].

The antimicrobial peptides (AMPs) of honey bees, mainly including abaecin, apidaecin, defensin, and hymenoptaecin, are important to their humoral immune systems, being regulated by the Toll and Imd/JNK signaling pathways [68,69]. These small-molecule peptides, encoded by specific genes, exhibit diverse antibacterial spectra and can effectively inhibit the growth and development of invading microbial pathogens. They consequently play a pivotal role in the development and resilience of bees when facing external stress [70], and variations in the expression levels of AMP genes have a direct impact on the sensitivity of workers to pathogens [71]. The expression levels of four AMP genes (*Aba*, *Api*, *Def1*, *Hym*) were increased in the 1-day-old workers but decreased in the 21-day-old workers (Figure 4), which may be related to the different physiological statuses of workers of different ages [16,49,72]. The 1-day-old workers have a more sensitive physiological stress response system and can produce a stronger immune response to azoxystrobin exposure than the 21-day-old workers [73]. This response rapidly activates AMP gene expression to produce more antimicrobial peptides to counteract the potential damage from the pesticides and maintain health [74]. However, this in turn affects the corresponding synthetic and metabolic pathways, such as the Toll and Imd/JNK signaling pathways, thereby affecting the overall stability and balance of their physiological state and altering their ability to adapt to the external environment [16,69,72]. In addition, the reduced expression of the four immune genes in the 21-day-old workers leads to a decrease in their immunity, which in turn increases their sensitivity to pathogens, with low survival rates, and even leads to food shortage and colony collapse, which is consistent with the previous findings on the survival rates of workers (Figure 1). The antimicrobial peptide abaecin exhibits a strong inhibitory effect against Gram-negative bacteria [69]. *Aba* expression was significantly reduced in the 8-day-old workers in the 250 mg/L group, potentially leading to a decreased resistance to Gram-negative bacteria. However, the upregulation of the other three immune genes (*Api*, *Def1*, *Hym*) could compensate for this deficiency and help maintain the workers’ resistance to pathogens [9,16,74].

The insulin/insulin-like signaling pathway (IIS) in honey bees directly influences their growth, development, metabolic balance, lifespan, and reproductive function [75,76]. It regulates sugar and lipid metabolism, protein synthesis, and cell proliferation and differentiation, and is involved in immune response and stress adaptation by activating molecules downstream of the target of the rapamycin (TOR) pathway [77,78,79]. Both *Iip1* and *Iip2* are expressed in the honey bee fat body, with *Ilp1* being related to lipoprotein synthesis and *Ilp2* reflecting nutrient status [80,81]. Despite their distinct roles, their functions are complementary, enabling them to integrate information on nutrient status and energy storage [45,82]. In the 21-day-old workers, the expression levels of both *Iip1* and *Iip2* decreased with increasing azoxystrobin concentrations (Figure 5), indicating that they had severe issues with the metabolism and storage of nutrients. This may be related to the damage produced to their midgut cells by azoxystrobin and the impact of this on their gut microbiota, which impacts nutrient absorption and metabolism [4,83]. However, *Iip1* and *Iip2* exhibited an increased expression in 1-day-old workers exposed to certain concentrations of azoxystrobin, which can be ascribed to the higher physiological plasticity of the 1-day-old workers, making them more sensitive to external stressors [84,85]. By rapidly upregulating the expression of *Iips*, they can regulate sugar and lipid metabolism, protein synthesis, and cell proliferation and differentiation, thereby enhancing their short-term metabolic adaptability [9,16,42,86]. In the 250 mg/L azoxystrobin treatment group, the 1-day-old workers exhibited a high *ilp1* expression, while the 21-day-old workers showed a low expression. This difference in expression patterns across ages may be attributed to variations in physiological condition and plasticity [16,41,49,83,84]. The slight increase in *ilp1* expression in the 1-day-old workers at 250 mg/L may indicate an attempt to compensate for metabolic stress through enhanced insulin signaling, and the 21-day-old workers may have reduced physiological plasticity and a lower capacity to upregulate *ilp1* in response to azoxystrobin stress, possibly due to cumulative damage or senescence [16,49,83]. Meanwhile, this may link to the sublethal effects of azoxystrobin on the fat body of workers, where *ilp1* is primarily expressed [41,83]. Additionally, the downregulation of *ilp1* in the 21-day-old workers might be a metabolic trade-off, where resources are diverted to other stress-response pathways, or due to the bees’ limited ability to cope with prolonged exposure [2,85]. Vitellogenin (Vg) is a pleiotropic and multifunctional protein in bees, involved in diverse physiological processes including climate adaptation, ovarian activation, reproductive competition, labor differentiation, behavior formation, and lifespan extension [87,88]. The results of this study confirm that azoxystrobin stress disrupts the nutritional assimilation and immune defense of workers. Azoxystrobin can induce an increase in the expression levels of *Vg* in workers of different ages, indicating that *Vg* is involved in the metabolic regulation and immune response of workers and plays a compensatory protective role [89,90]. Furthermore, *Vg* functions as a potent antioxidant capable of scavenging free radicals and mitigating oxidative damage resulting from azoxystrobin exposure, and this action helps to maintain physiological homeostasis and prolong lifespan [91,92].

## 5. Conclusions

Azoxystrobin exposure has significant adverse effects on honey bees, affecting their survival rates, enzyme activities, and gene expression profiles. The age-dependent variations in sensitivity emphasize the significance of taking into account the developmental stage of workers when assessing the impacts of agricultural chemicals on bee colonies. The findings suggest that azoxystrobin can disrupt the normal physiological functions of honey bees, potentially leading to decreased colony health and stability. More research is necessary to fully understand the long-term effects of azoxystrobin on honey bee colonies and to develop strategies for minimizing these impacts.

## Data Availability

The original contributions presented in this study are included in the article/Supplementary Materials. Further inquiries can be directed to the corresponding author.

## Supplementary Materials

The following supporting information can be downloaded at: https://www.mdpi.com/article/10.3390/insects16050449/s1, Table S1: Primers of development, nutrition, and immune genes used for qPCR. Reference [93] is cited in the Supplementary Materials.
