# Supplementary material for: Azoxystrobin-Induced Physiological and Biochemical Alterations in Apis mellifera Workers of Different Ages
Source: Insects. 2025 Apr 24;16(5):449. doi: 10.3390/insects16050449 (PMC12111993; doi:10.3390/insects16050449)
Supplement: Supplementary file 1 [file insects-16-00449-s001.zip › insects-3556763-supplementary.pdf]

Supplementary material

Primers of development, nutrition and immune genes used for qPCR

Table S1 Primers of development, nutrition and immune genes used for qPCR

Genes		Primer sequence (5'→3')[a]	Gene ID	Reference
Reference gene	*actin*	F: TTGTATGCCAACACTGTCCTTT<br>R: TGGCGCGATGATCTTAATTT	NM_001185145.1	Simone et al. (2009)
Immunity-related genes	abaecin (*Aba*)	F: CAGCATTCGCATACGTACCA<br>R: GACCAGGAAACGTTGGAAAC	NM_001011617.1	
	apidaecin (*Api*)	F: TTTTGCCTTAGCAATTCTTGTTG<br>R: GTAGGTCGAGTAGGCGGATCT	NM_001011613.1	Evans et al. (2006)
	defensin1 (*Def1*)	F: TGCGCTGCTAACTGTCTCAG<br>R: AATGGCACTTAACCGAAACG	NM_001011616.2	
	hymenoptaecin (*Hym*)	F: CTCTTCTGTGCCGTTGCATA<br>R: GCGTCTCCTGTCATTCCATT	NM_001011615.1	
Nutrient-related genes	insulin-like peptide 1 (*Ilp1*)	F: TGGCAAGGTGTCTATCACCG<br>R: ACGTCAGCAGCATATCACCA	XM_026442143.1	de Azevedo and Hartfelder (2008)
	insulin-like peptide 2 (*Ilp2*)	F: TTCCAGAAATGGAGATGGATG<br>R: TAGGAGCGCAACTCCTCTGT	NM_001177903.1	
	vitellogenin (*Vg*)	F: AGTTCCGACCGACGACGA<br>R: TTCCCTCCCACGGAGTCC	NM_001011578.1	Simone et al. (2009)

F, forward primer; R, reverse primer

Reference

Evans, J.D.; Aronstein, K.; Chen, Y.P.; Hetru, C.; Imler, J.L.; Jiang, H.; Kanost, M.; Thompson, G.J.; Zou, Z.; Hultmark, D. Immune pathways and defence mechanisms in honey bees *Apis mellifera*. *Insect Mol. Bio.* **2006**, *15*, 645–656.

de Azevedo, S. V.; Hartfelder, K. The insulin signaling pathway in honey bee (*Apis mellifera*) caste development-differential expression of insulin-like peptides and insulin receptors in queen and worker larvae. *J. Insect Physiol.* **2008**, *54(6)*, 1064–1071.

Simone, M.; Evans, J. D.; Spivak, M. Resin collection and social immunity in honey bees. *Evolution* **2009**, *63*, 3016–3022.
